# Supplementary material for: Exploring social vulnerability in National Health Safety Network surgical site infections
Source: Infect Control Hosp Epidemiol. 2025 Mar 26;46(6):589–96. doi: 10.1017/ice.2025.52 (PMC12169954; doi:10.1017/ice.2025.52)
Supplement: Dewitt et al. supplementary material 1 — Dewitt et al. supplementary material [file S0899823X25000522sup001.pdf]

Supplementary Table 1: Patient Characteristics Stratified by Social Vulnerability Index Quartiles

	Overall Social Vulnerability Index Quartiles								
	**1st** N = 6,178 (26%)		**2nd** N = 6,711 (28%)		**3rd** N = 5,686 (24%)		**4th** N = 5,193 (22%)		**p-value**[a]
**Demographics**									
Median Age, (IQR)	66	(54–74)	65	(53–73)	63	(51 – 72)	62	(49–71)	<0.01
Gender, n (%)									
Male	2,238	(36.2)	2,388	(35.5)	2,001	(35.2)	1,785	(34.4)	0.2
Race, n (%)									<0.01
White	5,073	(82.1)	5,436	(81.0)	4,307	(75.7)	2,855	(55.0)	
Black	711	(11.5)	913	(13.6)	1,032	(18.1)	1,874	(36.1)	
Hispanic	142	(2.3)	153	(2.3)	175	(3.1)	272	(5.2)	
Other	229	(3.7)	185	(2.8)	154	(2.7)	168	(3.2)	
Unknown	23	(0.4)	24	(0.4)	18	(0.3)	24	(0.5)	
**Region, n (%)**									<0.01
Charlotte Metropolitan	4,755	(77.0)	4,012	(59.8)	3,030	(53.3)	2,682	(51.6)	
Northern Georgia	129	(2.1)	326	(4.9)	396	(7.0)	362	(7.0)	
Central Georgia	526	(8.5)	644	(9.6)	775	(13.6)	875	(16.8)	
Greater Winston	768	(12.4)	1,729	(25.8)	1,485	(26.1)	1,274	(24.5)	
**Clinical Characteristics**									
Diabetes, n (%)	1,019	(16.5)	1,296	(19.3)	1,178	(20.7)	1,226	(23.6)	<0.01
Median BMI, (IQR)	29	(25–34)	30	(26–35)	30	(26–35)	31	(26–36)	<0.01
Trauma, n (%)	102	(1.7)	206	(3.1)	201	(3.5)	192	(2.7)	<0.001
**Procedure Characteristics**									
Number of Procedures									0.03
1	5,462	(88.4)	5,869	(87.5)	4,973	(87.5)	4,548	(87.6)	
2	644	(10.4)	747	(11.1)	644	(11.3)	573	(11.1)	
3	51	(0.8)	87	(1.3)	57	(1.0)	54	(1.0)	
4+	21	(0.3)	8	(0.1)	12	(0.2)	18	(0.3)	
Procedure Type, n (%)									<0.01
Total Knee Arthroplasty	1,816	(29.4)	1,975	(29.4)	1,658	(29.2)	1,370	(26.4)	
Total Hip Arthroplasty	1,442	(23.3)	1,526	(22.7)	1,304	(22.9)	1,113	(21.4)	
Colon	659	(10.7)	848	(12.6)	735	(12.9)	775	(14.9)	
Abdominal Hysterectomy	1,175	(19.0)	1,300	(19.4)	1,087	(19.1)	1,079	(20.8)	
Spinal Fusion	1,086	(17.6)	1,062	(15.8)	902	(15.9)	856	(16.5)	
Median Procedure Duration, minutes (IQR)	104	(81–144)	102	(80–144)	104	(80–147)	106	(81–154)	0.01
Emergency procedure, n (%)	168	(2.7)	269	(4.0)	284	(5.0)	275	(5.3)	<0.001
**Surgical Site Infection, n (%)**									
Surgical Site Infection	83	(1.3)	122	(1.8)	119	2.1	116	2.2	0.002

IQR Interquartile range; BMI Body mass index

[a] Wilcoxon rank sum test; T-test, Pearson's Chi-squared test; Fisher's exact test
