# Supplementary material for: Exploring social vulnerability in National Health Safety Network surgical site infections
Source: Infect Control Hosp Epidemiol. 2025 Mar 26;46(6):589–96. doi: 10.1017/ice.2025.52 (PMC12169954; doi:10.1017/ice.2025.52)
Supplement: Dewitt et al. supplementary material 2 — Dewitt et al. supplementary material [file S0899823X25000522sup002.pdf]

Supplementary Figure 1: Stratified Analysis of Racial/Ethnic Differences in Surgical Site Infection (SSI) Prevalence Across Social Vulnerability Index (SVI) Quartiles

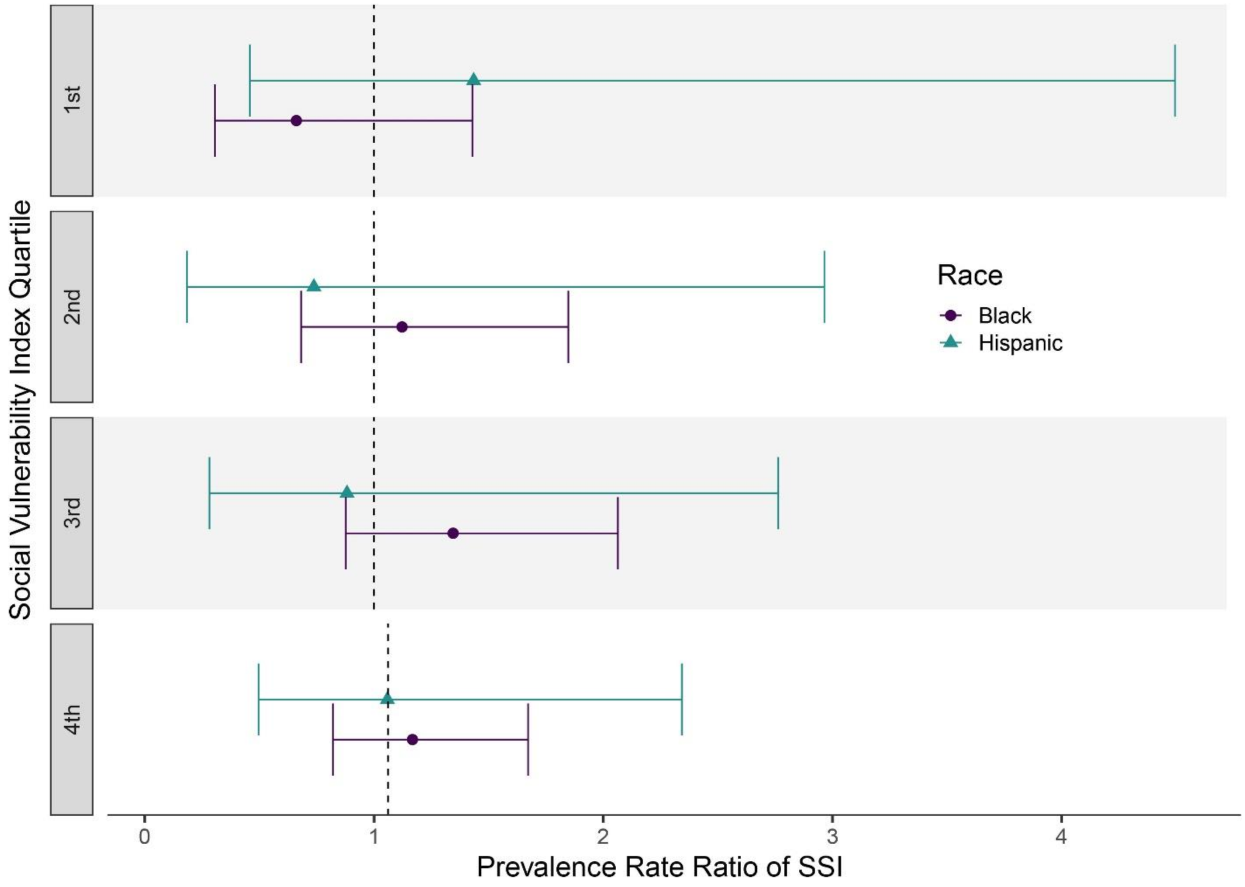

Supplementary Figure 1:

Estimated prevalence rate ratios of SSI that is stratified by SVI quartile with 95% confidence intervals shown. The reference group are those patients who indicated White race/ethnicity.
