# Supplementary material for: Exploring social vulnerability in National Health Safety Network surgical site infections
Source: Infect Control Hosp Epidemiol. 2025 Mar 26;46(6):589–96. doi: 10.1017/ice.2025.52 (PMC12169954; doi:10.1017/ice.2025.52)
Supplement: Dewitt et al. supplementary material 3 — Dewitt et al. supplementary material [file S0899823X25000522sup003.docx]

**Exploring Social Vulnerability in National Health Safety Network (NHSN) Surgical Site Infections**

Michael E. Dewitt MS^1,2^, Caroline E. Reinke MD MSHP^3^, Michael Inman BA^4^, Werner Bischoff MD PhD^1,5^, Shelley Kester MHA, BSN, CIC^5^, Anupama Neelakanta MD MPH^1,5^, Mindy M. Sampson DO^6^, Catherine L. Passaretti MD^1,5^

1. Section on Infectious Diseases, Department of Internal Medicine, Wake Forest University School of Medicine, Winston-Salem, NC, USA

2. Department of Biology, Wake Forest University, Winston-Salem, NC, USA

3. Department of Surgery, Atrium Health, Charlotte, NC, USA

4. Division of Business Intelligence and Data Analytics, Atrium Health, Charlotte, NC, USA

5. Department of Infection Prevention, Advocate Health, Charlotte, NC, USA

6. Division of Infectious Diseases & Geographic Medicine, Department of Medicine, Stanford University

**Corresponding author**:

Catherine Passaretti, MD,

1000 Blythe Blvd, Charlotte, NC 28203.

Office: 704-355-2459. Fax: 704 333-8984.

Email: [cpassare@wakehealth.edu](mailto:cpassare@wakehealth.edu)

**Prior Presentation:** Portions of this analysis were presented as an oral presentation at the Society of Healthcare Epidemiology of America Spring Conference in Houston, Texas in April 2024

**Abbreviated title**: Social Vulnerability Index and NHSN SSIs

**Key Words:** Social Vulnerability Index, Surgical Site Infections, Health Equity, Healthcare Associated Infections

**Word Count: 2885**

**Abstract (247 words, limit 250 words):**

Objective:
To assess the association between Social Vulnerability Index (SVI) and surgical site infections (SSIs) using National Healthcare Safety Network (NHSN) criteria.

Design:
Retrospective cohort study between August 1, 2022, and August 31, 2023.

Setting:
20 acute care hospitals in the Southeast United States

Patients:
23,768 total hip arthroplasty, total knee arthroplasty, abdominal hysterectomy, colon, and spinal fusion surgeries in 22,239 patients were included. Procedures with infection present at the time of surgery (PATOS) or incomplete geographic tracking data were excluded.

Methods:
Patient addresses as noted in the electronic health record were geocoded to determine census tract of residence and determine SVI. Demographic and clinical data were linked with SVI scores. SSIs were identified according to NHSN criteria. SVI was categorized into quartiles, and logistic regression was used to evaluate the association between SVI quartile (overall and for each SVI theme) and SSI risk. Subgroup analyses by procedure type and race were performed. Multivariable models of the association between overall SVI and SSI were adjusted for demographic and clinical factors.

Results:
Patients in the top SVI quartiles had significantly higher odds of developing SSIs after adjusting for other clinical and demographic factors. Increased risk was found for socioeconomic status and household characteristics themes, but not for the racial/ethnic minority theme. Association between SVI and SSI risk varied by type of surgery.

Conclusions:
Living in an area with a higher SVI is associated with increased SSI risk. Targeted interventions are needed to mitigate these disparities and improve outcomes.

**Introduction**

Surgical site infections (SSIs) represent a significant challenge in the realm of surgical care and infection prevention. Approximately 1-3% of surgical patients develop an SSI which can lead to prolonged hospital stays, more surgeries, increased mortality and care costs up to 3 times higher than for patients without an SSI​​.^1^ The social vulnerabilities contributing to SSIs are complex and multifaceted, involving both individual and community-level determinants.

The Social Vulnerability Index (SVI), developed by the Centers for Disease Control and Prevention (CDC) and the Agency for Toxic Substances and Disease Registry (ATSDR), is a composite measure initially designed to identify communities needing support before, during, and after disasters.^2^ This index incorporates 16 US Census variables which are compiled into four themes: 1) socioeconomic status, 2) household characteristics, 3) racial and ethnic minority status and 4) housing type and transportation. Higher SVI scores indicate greater vulnerability. The SVI provides a standardized approach for assessing the social determinants that impact health outcomes and can help identify systemic drivers of disparities that extend beyond individual risk factors.

Several studies have shown an association between various social vulnerability indices, and surgical complications.^3-6^ Post-operative patients who live in areas with higher vulnerability indices have increased post-surgical readmission rates, longer lengths of stay and higher mortality rates.^3,4^ Several studies have suggested that patients from areas with higher Area Deprivation Indices (ADI) who underwent hemiarthroplasty or total knee arthroplasty had higher rates of surgical site infections when compared to their peers from areas with lower ADI.^7,8^ Furthermore, Dyas et al. demonstrated that social vulnerability is linked to higher risk-adjusted rates of postoperative complications, including infection, even after risk adjustment across a broad surgical population​​.^9^ However, these studies showing an association between SVI and infection have largely used coded data to identify infected patients which has lower sensitivity and positive predictive value when compared to SSIs identified by trained infection preventionists utilizing National Health Safety Network (NHSN) definitions.^10-12^

Although research is limited, current evidence suggests there may be differences in the incidence of SSIs between racial groups in some surgeries and settings.^13,14^ A study of over 740,000 patients in the National Surgical Quality Improvement Program (NSQIP) database found racial differences in SSI rates across various surgical subspecialties, with Black patients experiencing higher rates of SSIs following vascular, orthopedic, and gynecological surgeries, compared to Non-Hispanic White patients​​.^15^ While differences in medical outcomes by race have been documented,^16^ race and social vulnerability are inexorably linked. Systemic racism underpins many of the socioeconomic and household factors captured by vulnerability indices, including the SVI. As a result, race is heavily correlated with external conditions such as socioeconomic barriers, healthcare access limitations, and the prevalence of certain comorbidities.^17-19^

Our study aims to evaluate the association between social vulnerability and surgical site infections utilizing NHSN criteria across 5 types of surgical procedures. To our knowledge this is the first study evaluating NHSN SSI outcomes and the CDC SVI metric.

**Methods**

This retrospective cohort study was conducted between August 1, 2022, and August 31, 2023, across 20 facilities in four geographically distinct regions: the Charlotte Metropolitan and Greater Winston areas in North Carolina, North Georgia and Central Georgia areas. We included patients who underwent one or more of the following procedures: total knee arthroplasty, total hip arthroplasty, abdominal hysterectomy, colon, or spinal fusion surgery. Surgical procedures of interest were identified using NHSN operative procedure codes.^12,20^

Patient residential addresses as documented in the electronic health record were geocoded to determine census tract and the designated SVI score. Procedures for patients with low-confidence Geographic Information System (GIS) data – such as those listing a post office box, incomplete or missing address, or indicating homelessness – were excluded from the analysis to ensure accuracy and consistency of the dataset used for SVI calculations and avoid misclassification,.

Demographic and key clinical characteristics associated with increased risk of SSI, such as procedure type, procedure duration,^21^ urgency of the procedure,^22^ body mass index (BMI),^23^ and a diagnosis of diabetes^24^ were extracted from the medical record to match the time of the procedure and merged with SVI data.

SSIs were identified by trained infection preventionists using National Healthcare Safety Network (NHSN) definitions^20^ and linked to surgical encounters. Procedures identified to have an SSI that met NHSN criteria for infection present at the time of surgery (PATOS) were excluded from the analysis.

Descriptive statistics including Wilcoxon rank sum test, T-test, Pearson's Chi-squared test, Fisher's exact test were used to describe and compare characteristics in patients with and without SSI. Overall and theme SVI were divided into quartiles with the odds of SSI in the highest SVI quartiles compared to the lower quartiles. Subgroup analyses were conducted by procedure type.

Patient characteristics were stratified by SVI quartiles to identify potential confounding factors associated with both higher SVI and SSI which were then excluded from the multivariable analysis.

We used thin-plate smoothing splines in a Bayesian hierarchical logistic regression model to account for the nonlinear response in SVI and the likelihood of SSI. The multivariable model was adjusted for region and multiple procedures using random effects and further adjusted for patient-level characteristics, including age, gender, and procedure type.

Patient race and ethnicity were determined by the patient’s report as documented in the medical record at the time of registration. To assess for potential differences within racial and ethnic groups, we generated two models using Poisson regression with robust standard errors. The first model calculated the prevalence rate ratio of SSI for each SVI quartile, using the first SVI quartile as the reference, separately for White, Black, and Hispanic patients. The second model calculated the prevalence rate ratio of SSI for Black and Hispanic patients, using White patients as the reference group, separately within each SVI quartile.

In all cases we used the “mgcv” R package with the package default priors.^25^

All analysis was completed in R version 4.4.1. The Wake Forest University Institutional Review Board approved the study.

**Results**

A total of 24,990 surgical procedures were included. Approximately 5% of procedures in the study population were excluded due to patients having either low confidence GIS addresses or meeting NHSN criteria for an SSI with infection present at the time of surgery. The remaining 23,768 procedures on 22,239 patients were included in the study. Twelve percent of procedures were performed on patients that had more than one procedure during the study period. The overall SSI rate was 2% (n=536), with the highest rates observed after colon surgery (5.8%) and abdominal hysterectomy (1.8%). (Figure 1).

The majority of procedures occurred in the Charlotte metropolitan area (61%). The patient population was predominantly white (74%) with a median age of 64. 4719 procedures (20%) were performed on patients with a preexisting diagnosis of diabetes and the median body mass index (BMI) of patients undergoing procedures was 30 kg/m^2^. 5229 procedures (22%) occurred in patients living in areas with an SVI in the fourth quartile. The median SVI was lower for the racial and ethnic minority status (0.45) and housing type and transportation themes (0.43) than that for the household characteristics (0.48) and socioeconomic status themes (0.50). This suggests a relatively higher level of vulnerability in the themes of household characteristics and socioeconomic status in our patient population. Patients who underwent a procedure complicated by SSI had significantly higher median overall SVI compared to those without SSI. When evaluating the SVI themes, all themes except for racial and ethnic minority status showed a similar association between higher median SVI and risk of SSI. (Table 1)

There was a relatively equal distribution of procedures on patients amongst SVI quartiles for all procedure types. Higher BMI, a diagnosis of diabetes, longer procedure duration and undergoing an emergent or trauma-related procedure were associated with both SSI (Table 1) and living in areas with higher SVI quartiles. (Supplementary Table 1)

In the unadjusted analysis, the odds of developing an SSI were significantly and progressively higher for procedures performed on patients from areas in higher overall SVI quartiles compared to the lowest. Notably, when evaluating individual SVI themes, patients in the fourth quartile for socioeconomic and household characteristic themes had increased odds of SSI compared to those in the first quartile. A similar association was not seen for the racial and ethnic minority status theme. The association between SVI and SSI in the housing type and transportation theme was more variable, with the third quartile, but not the fourth, being associated with a significantly increased risk of SSI compared to the first. (Figure 2)

In the multivariable model adjusting for geographic region, age, gender, procedure type, and multiple procedures, the association between higher overall SVI and increased odds of SSI remained significant (p=0.04). The relationship between SVI and SSI was found to be nonlinear, with the increasing odds of SSI plateauing at SVI greater than 0.75 (Figure 3).

In the models stratified by race and ethnic groups, higher SVI quartiles were associated with a higher prevalence of SSI among both Black and White patients, although the effect was more pronounced in Black patients. (Figure 4) When we stratified by SVI quartile to compare across race and ethnic groups, we did not observe a significant difference in SSI rates between Black or Hispanic patients and White patients. (Supplementary Figure 1)

In the subgroup analysis by surgery type, the association between overall SVI score and the odds of SSI was strongest for patients who underwent spinal fusion or total knee arthroplasty procedure. Spinal fusion procedures on patients in the highest SVI quartile were 2.2 times more likely to be complicated by SSI compared to those on patients in the lowest SVI quartile. (95% CI 1.1 – 4.5, p=0.03). Although there was a trend toward increased SSI risk among total knee arthroplasty procedures performed on patients from the fourth SVI quartile compared to the first, this difference did not reach statistical significance. However, knee arthroplasty procedures performed on patients in the third SVI quartile were 3.3 times more likely to be complicated by an SSI compared to those in the first SVI quartile (95% CI: 1.3 – 8.3, p = 0.01). Similar, though non-significant, trends were observed for the other surgery types, with the exception of hip arthroplasty (Figure 5).

**Discussion**

Although this is the first study to examine the relationship between NHSN-defined surgical site infections (SSI) and the Social Vulnerability Index (SVI), several studies in the literature have identified associations between measures of social vulnerability and coded data on postoperative infections and other surgical outcomes.^5,6,15,26-28^ The findings of this study underscore the significant association between social vulnerability and postoperative SSIs. Patients residing in areas with high social vulnerability, as measured by the SVI, are at an increased odds of developing an SSI that meets NHSN criteria, even after accounting for individual demographic and clinical characteristics.

Interestingly, we observed an increased risk of SSI among patients residing in areas with higher SVI quartiles for socioeconomic status and household characteristics, but this association was not evident in the race and minority status SVI theme. It is important to note that systemic racism underlies many of the socioeconomic and household characteristics captured by the SVI. Although higher race and minority status theme SVI was not significantly associated with SSI in our analysis, this does not indicate that racism is unimportant. Instead, it reflects how structural racism manifests in broader societal factors, such as income inequality, inadequate housing, and reduced access to healthcare resources, which are measured in other SVI domains. Notably when we stratified by race, we did observe an increased SSI risk in the higher SVI quartiles for both Black and White patients, but the effect was more pronounced in Black patients.

The underlying causes for the observed association between living in an area with high social vulnerability and SSI outcomes are undoubtedly multifactorial. Individuals from socially vulnerable backgrounds often face barriers to accessing timely and equitable healthcare, including preventive measures and presumably postoperative care.^29^ Socioeconomic disadvantages can limit access to clean living conditions, proper nutrition and necessary medical resources—all of which are critical for optimal recovery.^29-31^ Transportation challenges may limit ability to follow-up postoperatively.^32^ Additionally, social vulnerability is frequently associated with higher rates of comorbidities such as diabetes^33^ and obesity^33,34^, which can impact the risk of developing a SSI^35^. Our data confirm that patients that live in areas with higher SVI scores often have higher BMI, a greater incidence of diabetes, more emergent procedures and longer procedure durations—all factors that may contribute to poorer surgical outcomes.^35^ Limited health literacy^36^ and diminished trust in healthcare providers^37^ can further complicate adherence to postoperative instructions and follow-up care, also impacting the likelihood of infection. Addressing these disparities requires a multifaceted approach that includes improving access to healthcare, enhancing patient education and providing targeted support to socially vulnerable populations to reduce the risk of SSIs.

We observed a relatively linear increase in SSI risk among individuals living in areas with low to moderately high SVI scores; however, the risk plateaued for those procedures performed on patients living in areas with SVI scores above 0.75. Several drivers may explain this phenomenon. Biological and behavioral risk factors may plateau at more extreme levels of social vulnerability and as such their additional contribution to SSI risk may level off. At the highest levels of social vulnerability, other factors, such as hospital-level characteristics (e.g., resource availability, infection control practices, postoperative care delivery) may play more of a role. Patients living in areas with high SVI may be disproportionately lost to follow-up or less likely to undergo elective surgeries due to barriers in healthcare access, leaving only those with urgent or life-threatening conditions, where infection risk may be driven more by the nature of the surgery than by social vulnerability. Finally, those patients living in areas with the highest SVI scores may face uniformly high levels of multiple risk factors leading to a “ceiling effect” (i.e. once a certain threshold of vulnerability is reached, additional increases in SVI might not significantly escalate risk because these individuals are already maximally impacted).

In the subgroup analysis for overall SVI and type of procedure, we found some variability in results. This finding bears future investigation to determine whether these differences reflect variations in procedure-specific risk profiles (e.g., lower baseline risk of infection) and potentially unmeasured confounders such as access to postoperative care, patient selection bias, or more advanced pre- and post-operative optimization protocols. We did not analyze individual types of procedure by SVI themes, so it is also possible that different SVI themes had varying impact on different types of surgeries.

To effectively address the detected disparities and enhance patient care for those from areas with higher social vulnerability, a multifaceted approach is needed. Prospective identification of patients at increased risk due to social vulnerability may allow for targeted use of preoperative optimization strategies to optimize patients’ health before surgery. Interventions include nutritional support, glycemic control, weight loss and smoking cessation as examples.^1^ In addition, providing additional resources and support postoperatively may have an even more sustained impact for patients from high SVI areas. Ensuring access to follow-up care, home health services and targeted education on wound care and infection prevention could be considered. Access to community health workers, social workers and patient navigators can play a vital role in connecting patients with necessary services and support systems.^38,39^ Healthcare systems should advocate for policies that address the structural factors that feed into social vulnerability including supporting initiatives aimed at increasing access to healthcare services in underserved communities. Ongoing research is essential to understand the complex interplay of social and clinical factors affecting surgical outcomes.

One of the study's strengths is its large sample size and inclusion of multiple geographic regions, which enhances the generalizability of the findings. However, variations in local demographics, healthcare infrastructure and region-specific factors can influence SSI risk. While similar patterns between SVI and SSI may emerge, further studies are needed in diverse settings. Several other limitations should be acknowledged beyond the retrospective nature of the study. The exclusion of patients with low-confidence addresses may introduce selection bias and underrepresent vulnerable groups such as individuals living with homelessness. By removing these records, the analysis may underestimate true infection rates among more vulnerable communities. Similarly, the addresses which were used were those that were documented in the EHR, and do not reflect changes in address over the duration of care. Finally, the complex interplay of social and clinical factors may not be fully captured by the SVI alone (e.g., SVI reflects community-level rather than individual-level social determinants of health).

In conclusion, this study underscores the profound impact of social vulnerability, as captured by the SVI, on the risk of SSI. While race alone did not emerge as a direct predictor in our analysis, systemic racism reflected through socioeconomic and household vulnerabilities, remains a fundamental driver behind health disparities in surgical outcomes. This finding calls for a shift in how we approach disparities in SSI risk, moving beyond the use of race as a determinant, to focus on addressing the structural inequities that disproportionately affect marginalized communities. Targeted interventions that address social vulnerability through community level support, enhanced access to resources and healthcare equity, are crucial for mitigating the risk of SSIs and improving health outcomes for all patients. As healthcare systems work toward reducing inequities, addressing the sources of social vulnerability will be imperative in ensuring more effective, equitable and sustainable improvements in postoperative care.

**Acknowledgements:**

*Financial Support*. None reported.

*Potential conflicts of interest.* All authors report no conflicts of interest relevant to this article.

**References**

**1.** Calderwood MS, Anderson DJ, Bratzler DW, et al. Strategies to prevent surgical site infections in acute-care hospitals: 2022 Update. *Infect Control Hosp Epidemiol* 2023;44:695-720.

**2.** Flanagan BEG, E. W.; Hallisey, E. J.; Heitgerd, J. L.; Lewis, B. A Social Vulnerability Index for Disaster Management. *Journal of Homeland Security and Emergency Management* 2011;8.

**3.** Paro A, Hyer JM, Diaz A, Tsilimigras DI, Pawlik TM. Profiles in social vulnerability: The association of social determinants of health with postoperative surgical outcomes. *Surgery* 2021;170:1777-1784.

**4.** Ghirimoldi FM, Schmidt S, Simon RC, et al. Association of Socioeconomic Area Deprivation Index with Hospital Readmissions After Colon and Rectal Surgery. *J Gastrointest Surg* 2021;25:795-808.

**5.** Abdelhack M, Tripathi S, Chen Y, Avidan MS, King CR. Social vulnerability and surgery outcomes: a cross-sectional analysis. *BMC Public Health* 2024;24:1907.

**6.** Diaz A, Hyer JM, Barmash E, Azap R, Paredes AZ, Pawlik TM. County-level Social Vulnerability is Associated With Worse Surgical Outcomes Especially Among Minority Patients. *Ann Surg* 2021;274:881-891.

**7.** Baidya J, Gordon AM, Nian PP, et al. Social determinants of health in patients undergoing hemiarthroplasty: are they associated with medical complications, healthcare utilization, and payments for care? *Arch Orthop Trauma Surg* 2023;143:7073-7080.

**8.** Rahman TM, Shaw JH, Mehaidli A, et al. The Impact of Social Determinants of Health on Outcomes and Complications After Total Knee Arthroplasty: An Analysis of Neighborhood Deprivation Indices. *J Bone Joint Surg Am* 2024;106:288-303.

**9.** Dyas AR, Carmichael H, Bronsert MR, et al. Social vulnerability is associated with higher risk-adjusted rates of postoperative complications in a broad surgical population. *Am J Surg* 2024;229:26-33.

**10.** Jhung MA, Banerjee SN. Administrative coding data and health care-associated infections. *Clin Infect Dis* 2009;49:949-955.

**11.** van Mourik MS, van Duijn PJ, Moons KG, Bonten MJ, Lee GM. Accuracy of administrative data for surveillance of healthcare-associated infections: a systematic review. *BMJ Open* 2015;5:e008424.

**12.** Bucher BT, Yang M, Arndorfer J, et al. Changes in the accuracy of administrative data for the detection of surgical site infections. *Infect Control Hosp Epidemiol* 2021;42:1128-1130.

**13.** Ibrahim SA, Stone RA, Han X, et al. Racial/ethnic differences in surgical outcomes in veterans following knee or hip arthroplasty. *Arthritis Rheum* 2005;52:3143-3151.

**14.** Sanford Z, Taylor H, Fiorentino A, et al. Racial Disparities in Surgical Outcomes After Spine Surgery: An ACS-NSQIP Analysis. *Global Spine J* 2019;9:583-590.

**15.** Welter M, Grosh K, Jose J, et al. Are There Racial Differences in the Rate of Surgical Site Infection Based on Surgical Subspecialty? *Surg Infect (Larchmt)* 2023;24:860-868.

**16.** White TM, Borrell LN, El-Mohandes A. A Review of the Public Health Literature Examining the Roles of Socioeconomic Status and Race/Ethnicity on Health Outcomes in the United States. *J Racial Ethn Health Disparities* 2024.

**17.** Williams DR, Priest N, Anderson NB. Understanding associations among race, socioeconomic status, and health: Patterns and prospects. *Health Psychol* 2016;35:407-411.

**18.** Marcelin JR, Hicks LA, Evans CD, Wiley Z, Kalu IC, Abdul-Mutakabbir JC. Advancing health equity through action in antimicrobial stewardship and healthcare epidemiology. *Infect Control Hosp Epidemiol* 2024;45:412-419.

**19.** Fiscella K, Franks P, Gold MR, Clancy CM. Inequality in quality: addressing socioeconomic, racial, and ethnic disparities in health care. *JAMA* 2000;283:2579-2584.

**20.** NationalHealthcareSafetyNetwork. Surgical Site Infection (SSI) Events. 2024. <https://www.cdc.gov/nhsn/psc/ssi/index.html?CDC_AA_refVal=https%3A%2F%2Fwww.cdc.gov%2Fnhsn%2Facute-care-hospital%2Fssi%2Findex.html>. Accessed 11/25/2024.

**21.** Cheng H, Chen BP, Soleas IM, Ferko NC, Cameron CG, Hinoul P. Prolonged Operative Duration Increases Risk of Surgical Site Infections: A Systematic Review. *Surg Infect (Larchmt)* 2017;18:722-735.

**22.** Gagen B, Hall C. Preventing Surgical Site Infections in Emergency General Surgery: Current Strategies and Recommendations. *Current Surgery Reports* 2024;12:227-237.

**23.** Meijs AP, Koek MBG, Vos MC, Geerlings SE, Vogely HC, de Greeff SC. The effect of body mass index on the risk of surgical site infection. *Infect Control Hosp Epidemiol* 2019;40:991-996.

**24.** Martin ET, Kaye KS, Knott C, et al. Diabetes and Risk of Surgical Site Infection: A Systematic Review and Meta-analysis. *Infect Control Hosp Epidemiol* 2016;37:88-99.

**25.** Wood SN. *Generalized Additive Models: An Introduction with R.* Second Edition ed: Chapman and Hall/CRC; 2017.

**26.** Carmichael H, Dyas AR, Bronsert MR, et al. Social vulnerability is associated with increased morbidity following colorectal surgery. *Am J Surg* 2022;224:100-105.

**27.** Stuart CM, Dyas AR, Byers S, et al. Social vulnerability is associated with post-operative morbidity following robotic-assisted lung resection. *J Thorac Dis* 2023;15:5931-5941.

**28.** Stevens J, Reppucci ML, Pickett K, et al. Using the Social Vulnerability Index to Examine Disparities in Surgical Pediatric Trauma Patients. *J Surg Res* 2023;287:55-62.

**29.** Al Rifai M, Jain V, Khan SU, et al. State-Level Social Vulnerability Index and Healthcare Access: The Behavioral Risk Factor Surveillance System Survey. *Am J Prev Med* 2022;63:403-409.

**30.** Alkerwi A, Vernier C, Sauvageot N, Crichton GE, Elias MF. Demographic and socioeconomic disparity in nutrition: application of a novel Correlated Component Regression approach. *BMJ Open* 2015;5:e006814.

**31.** Jacobs DE. Environmental health disparities in housing. *Am J Public Health* 2011;101 Suppl 1:S115-122.

**32.** Goins RT, Williams KA, Carter MW, Spencer M, Solovieva T. Perceived barriers to health care access among rural older adults: a qualitative study. *J Rural Health* 2005;21:206-213.

**33.** Cleveland JC, 3rd, Espinoza J, Holzhausen EA, Goran MI, Alderete TL. The impact of social determinants of health on obesity and diabetes disparities among Latino communities in Southern California. *BMC Public Health* 2023;23:37.

**34.** Aris IM, Perng W, Dabelea D, et al. Associations of Neighborhood Opportunity and Social Vulnerability With Trajectories of Childhood Body Mass Index and Obesity Among US Children. *JAMA Netw Open* 2022;5:e2247957.

**35.** Korol E, Johnston K, Waser N, et al. A systematic review of risk factors associated with surgical site infections among surgical patients. *PLoS One* 2013;8:e83743.

**36.** Theiss LM, Wood T, McLeod MC, et al. The association of health literacy and postoperative complications after colorectal surgery: A cohort study. *Am J Surg* 2022;223:1047-1052.

**37.** World Health Organization. Adherence to long-term therapies : evidence for action. 2003. <https://iris.who.int/handle/10665/42682>.

**38.** Roland KB, Milliken EL, Rohan EA, et al. Use of Community Health Workers and Patient Navigators to Improve Cancer Outcomes Among Patients Served by Federally Qualified Health Centers: A Systematic Literature Review. *Health Equity* 2017;1:61-76.

**39.** Li HW, Scanlon ML, Kisilu N, Litzelman DK. The role of community health workers in the surgical cascade: a scoping review. *Hum Resour Health* 2021;19:122.

**Figure Legends**

**Figure 1:** This figure illustrates the study population, detailing the inclusion and exclusion criteria as well as detailing the type and number of procedures with the corresponding surgical site infection (SSI) rates expressed as the number of SSIs per 100 procedures.

**Figure 2:** This figure compares the unadjusted odds of surgical site infection (SSI) across social vulnerability index (SVI) quartiles for all procedures combined. For overall SVI and individual SVI themes, odds for each quartile with the associated 95% confidence intervals are presented relative to the lowest SVI quartile as the reference group.

**Figure 3**: This figure illustrates the odds of surgical site infection (SSI) by Social Vulnerability Index (SVI) score, after adjusting for geographic region, multiple procedures, age, gender, and procedure type. Factors associated with both living in a top quartile SVI area and SSI risk were not included in the model to minimize confounding.

**Figure 4:** This figure illustrates estimated prevalence rate ratios of SSI for each overall SVI quartile stratified by racial/ethnic groups with 95% confidence intervals shown (error bars). Note the reference groups is the first SVI quartile (lowest social vulnerability)

**Figure 5**: This figure illustrates the unadjusted odds of surgical site infection (SSI) across social vulnerability index (SVI) quartiles stratified by type of procedure. For each type of surgery, odds for each quartile of overall SVI with the associated 95% confidence interval are presented relative to the lowest SVI quartile as the reference group.

**Table 1: Demographic and clinical characteristics of patients undergoing surgical procedures, comparing patients with and without surgical site infections**

|  | **All Procedures**  N = 23768 | | **No SSI**  N = 23328 | | **SSI**  N = 440 | | **p-value^a^** |
| --- | --- | --- | --- | --- | --- | --- | --- |
| **Demographics** | | | | | | | |
| Geographic Region, n (%)  Charlotte Metropolitan  Greater Winston  North Georgia   Central Georgia | 14479  5256  1213  2820 | (61) (22) (5) (12) | 14238 1190 2768 5132 | (61) (5) (12) (22) | 241 23 52 124 | (55) (5) (12) (28) | 0.02 |
| Median Age (IQR) | 64 | (52-73) | 64 | (51-72) | 60 | (46-71) | <0.01 |
| Male, n (%) | 8412 | (35) | 8235 | (35) | 177 | (40) | 0.03 |
| Race, n (%)     Non-Hispanic White     Non-Hispanic Black     Hispanic     Other/Unknown | 17671 4530 742 825 | (74) (19) (3) (3) | 17353 4432 728 815 | (74) (19) (3) (3) | 318 98 14 10 | (72) (22) (3) (2) | 0.27 |
| **Procedural Characteristics** | | | | | | | |
| Procedure Type, n (%)  Total Knee Arthroplasty  Total Hip Artroplasty  Colon  Abdominal Hysterectomy  Spinal Fusion | 6819 5385 3017 4641 3906 | (29) (23) (13) (20) (16) | 6768 5316 2841 4556 3847 | (29) (23) (12) (20) (17) | 51 69 176  85 59 | (12) (16) (40) (19) (13) | <0.01 |
| Procedure Count, n (%)  1  2  3+ | 20852  2608  308 | (88)  (11)  (1) | 20564  2494  270 | (88)  (11)  (1) | 288  114  38 | (65)  (26)  (9) | <0.001 |
| ASA Score, n (%)  1  2  3  4  5 | 368  8762  13116  1430  92 | (2) (37) (55) (6) (0) | 366 8656 12846 1373 87 | (2) (38) (55) (6) (0) | 2 106 270 57 5 | (1) (24) (61) (13) (1) | <0.01 |
| Trauma, n (%) | 701 | (3) | 671 | (3) | 30 | (7) | <0.01 |
| Emergency, n (%) | 996 | (4) | 941 | (4) | 55 | (13) | <0.01 |
| Median Procedure Duration,  minutes (IQR) | 104 | (80-147) | 103 | (80-147) | 148 | (98-215) | <0.01 |
| Median BMI (IQR) | 30 | (26-35) | 30 | (26-35) | 30 | (25-35) | 0.80 |
| Diabetes, n (%) | 4719 | (20) | 4602 | (20) | 117 | (27) | <0.01 |

| **Social Vulnerability Index Themes, median (IQR)** | | | | | | | |  |
| --- | --- | --- | --- | --- | --- | --- | --- | --- |
| Overall | 0.46 | (0.24-0.71) | 0.46 | (0.24-0.71) | 0.54 | (0.31-0.77) | <0.001 | |
| Socioeconomic Status | 0.50 | (0.29-0.75) | 0.50 | (0.29-0.75) | 0.56 | (0.36-0.79) | <0.001 | |
| Household Characteristics | 0.48 | (0.25-0.76) | 0.48 | 0.25-0.75) | 0.55 | (0.31-0.78) | 0.006 | |
| Racial and Ethnic Minority Status | 0.45 | (0.27-0.64) | 0.45 | (0.27-0.64) | 0.44 | (0.27-0.66) | 0.8 | |
| Housing Type and Transportation | 0.43 | (0.22-0.66) | 0.43 | (0.22-0.66) | 0.47 | (0.25-0.68) | 0.03 | |

| **SSI Characteristics, n (%)** | | | | |  |  |
| --- | --- | --- | --- | --- | --- | --- |
| Depth of Infection  Superficial  Deep/Organ Space | 144  296 | (0.6) (1.2) | --- | --- | | --- |

SSI, Surgical Site Infection; IQR, Interquartile range; ASA American Society of Anesthesiologists, BMI Body Mass Index

a Wilcoxon rank sum test; T-test, Pearson's Chi-squared test; Fisher's exact test
